# Supplementary material for: Identification of photoperiod-induced specific miRNAs in the adrenal glands of Sunite sheep (Ovis aries)
Source: Front Vet Sci. 2022 Jul 22;9:888207. doi: 10.3389/fvets.2022.888207 (PMC9354845; doi:10.3389/fvets.2022.888207)
Supplement: Supplementary file 1 [file Data_Sheet_1.ZIP › Supplementary file/miRDeep2 file.zip!/miRDeep2 file/Know_Structure/SPLP42A1/oar-mir-136.pdf]

miRBase precursor : oar-mir-136
Total read count : 8254
oar-miR-136 read count : 2226
remaining reads : 6028

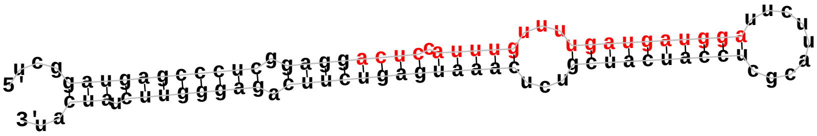

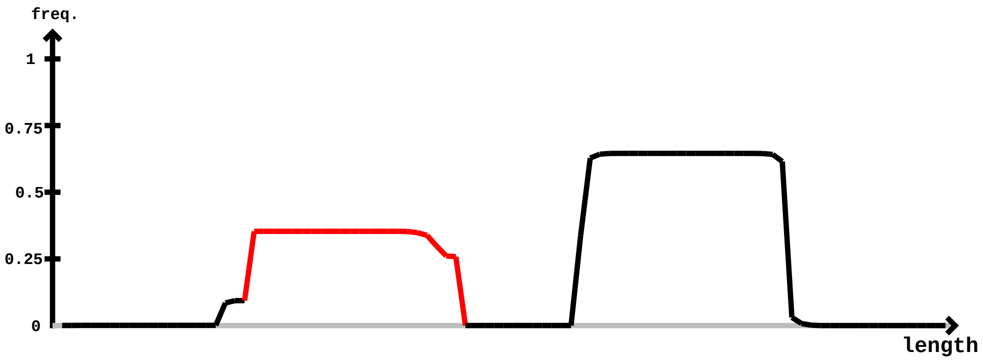


```
                                     oar-miR-136
5'- ucggaugagcccucggaggacuccauuuguuuugaugauggauucuuacgcuccaucaucgucucaaaugagucuucagaggguucuaucau   -3'    exp
    ...(((((((((((.((((((((.(((((...((((((((((.........))))))))))...))))))))))))).)))))))).)))..     reads       mm          sample
    ucggaugagcccucggagg.........................................................................     3           0           seq
    .cggaugagcccucggagg.........................................................................     4           0           seq
    ................aggacuccauuuguuuuga.........................................................     7           0           seq
    ................aggacuccauuuguuuugau........................................................     34          0           seq
    ................aggacuccauuuguuuugaug.......................................................     80          0           seq
    ................aggacuccauuuguuuugauga......................................................     323         0           seq
    ................aggacuccauuuguuuugaugau.....................................................     247         0           seq
    ................aggacuccauuuguuuugaugaug....................................................     1           0           seq
    .................ggacuccauuuguuuugau........................................................     2           0           seq
    .................ggacuccauuuguuuugaug.......................................................     2           0           seq
    .................ggacuccauuuguuuugauga......................................................     10          0           seq
    .................ggacuccauuuguuuugaugau.....................................................     55          0           seq
    .................ggacuccauuuguuuugaugaug....................................................     4           0           seq
    ...................acuccauuuguuuugaugau.....................................................     4           0           seq
    ...................acuccauuuguuuugaugaug....................................................     18          0           seq
    ...................acuccauuuguuuugaugaugg...................................................     2127        0           seq
    ...................acuccauuuguuuugaugaugga..................................................     1           0           seq
    ....................cuccauuuguuuugaugaugg...................................................     3           0           seq
    .........................................auucuuacgcuccaucaucgucucaaaugagucu.................     1           0           seq
    ..........................................uucuuacgcuccaucaucgucucaaaugagucu.................     1           0           seq
    .....................................................caucaucgucucaaaug......................     1           0           seq
    .....................................................caucaucgucucaaaugag....................     1           0           seq
    .....................................................caucaucgucucaaaugaA...................     2           1           seq
    .....................................................caucaucgucucaaaugagu...................     23          0           seq
    .....................................................caucaucgucucaaaugaguc..................     169         0           seq
    .....................................................caucaucgucucaaaugagucu.................     2565        0           seq
    .....................................................caucaucgucucaaaugagucuu................     24          0           seq
    ......................................................aucaucgucucaaaugagu...................     5           0           seq
    ......................................................aucaucgucucaaaugaguc..................     48          0           seq
    ......................................................aucaucgucucaaaugagucu.................     2222        0           seq
    ......................................................aucaucgucucaaaugagucuu................     116         0           seq
    ......................................................aucaucgucucaaaugagucuuc...............     7           0           seq
    .......................................................ucaucgucucaaaugaguc..................     1           0           seq
    .......................................................ucaucgucucaaaugagucu.................     33          0           seq
    .......................................................ucauUgucucaaaugagucu.................     1           0           seq
```

oar-miR-136

```
ucggaugagcccucggaggacuccauuuguuuugaugaugga uucuuacgcuccaucaucgucucaaaugagucuucagaggguucuaucau
.......................................................ucaucgucucaaaugagucuu................   47   0   seq
.......................................................ucaucgucucaaaugagucuuc...............   34   0   seq
.......................................................ucaucgucucaaaugagucuuca..............   4    0   seq
........................................................caucgucucaaaugagucu.................   1    0   seq
........................................................caucgucucaaaugagucuu................   1    0   seq
........................................................caucgucucaaaugagucuuc...............   11   0   seq
........................................................caucgucucaaaugagucuuca..............   8    0   seq
.........................................................aucgucucaaaugagucu.................   3    0   seq
```
